# Permanent Occlusion of Feeding Arteries and Draining Veins in Solid Mouse Tumors by Vascular Targeted Photodynamic Therapy (VTP) with Tookad

**DOI:** 10.1371/journal.pone.0010282

**Published:** 2010-04-22

**Authors:** Noa Madar-Balakirski, Catherine Tempel-Brami, Vyacheslav Kalchenko, Ori Brenner, David Varon, Avigdor Scherz, Yoram Salomon

**Affiliations:** 1 Department of Biological Regulation, The Weizmann Institute of Science, Rehovot, Israel; 2 Department of Veterinary Resources, The Weizmann Institute of Science, Rehovot, Israel; 3 Department of Plant Sciences, The Weizmann Institute of Science, Rehovot, Israel; 4 Department of Hematology, Hadassah-Hebrew University Medical Center, Jerusalem, Israel; University of Arizona, United States of America

## Abstract

**Background:**

Antiangiogenic and anti-vascular therapies present intriguing alternatives to cancer therapy. However, despite promising preclinical results and significant delays in tumor progression, none have demonstrated long-term curative features to date. Here, we show that a single treatment session of Tookad-based vascular targeted photodynamic therapy (VTP) promotes permanent arrest of tumor blood supply by rapid occlusion of the tumor feeding arteries (FA) and draining veins (DV), leading to tumor necrosis and eradication within 24–48 h.

**Methodology/Principal Findings:**

A mouse earlobe MADB106 tumor model was subjected to Tookad-VTP and monitored by three complementary, non-invasive online imaging techniques: Fluorescent intravital microscopy, Dynamic Light Scattering Imaging and photosensitized MRI. Tookad-VTP led to prompt tumor FA vasodilatation (a mean volume increase of 70%) with a transient increase (60%) in blood-flow rate. Rapid vasoconstriction, simultaneous blood clotting, vessel permeabilization and a sharp decline in the flow rates then followed, culminating in FA occlusion at 63.2 sec±1.5SEM. This blockage was deemed irreversible after 10 minutes of VTP treatment. A decrease in DV blood flow was demonstrated, with a slight lag from FA response, accompanied by frequent changes in flow direction before reaching a complete standstill. In contrast, neighboring, healthy tissue vessels of similar sizes remained intact and functional after Tookad-VTP.

**Conclusion/Significance:**

Tookad-VTP selectively targets the tumor feeding and draining vessels. To the best of our knowledge, this is the first mono-therapeutic modality that primarily aims at the larger tumor vessels and leads to high cure rates, both in the preclinical and clinical arenas.

## Introduction

The distinctive morphological and functional characteristics of tumor versus normal vasculature [1], [2], [3], together with its indispensable role in supporting tumor growth, render the tumor vasculature an attractive therapeutic target [4], [5]. Tumor vessels are often immature, permeable, highly fractured, architecturally disordered and lack external smooth muscle and pericyte support. In addition, the blood flow within demonstrates rheologic abnormalities, variable pressure and inconsistent flow rates that upset homeostasis [6], [7]. These features enable tumor targeting by both antiangiogenic therapy and vascular disrupting agents (VDA) which aim toward inhibition of neovessel recruitment [8] and destruction of established functional tumor microvessels [9], respectively. The relatively large, often tortuous feeding arteries (FA) and draining veins (DV), that transverse the tumor rim [10] typically remain functional, despite the above therapies and enable eventual tumor relapse. Consequently, these vessels, which comprise the tumor lifeline, provide a formidable therapeutic target for novel anti-vascular treatments such as vascular-targeted photodynamic therapy (VTP).

VTP generates a local burst of cytotoxic reactive oxygen species (ROS) upon photo-activation of a circulating sensitizing agent. Upon a single treatment session, the ROS impact results in complete tumor vascular destruction. The ultra-short lifetime of ROS confines their activity to the illuminated volume, sparing downstream tissues from their toxicity. This antivascular modality appears to exploit the disparate sensitivities of normal versus pathological vasculature to ROS. The heightened sensitivity of tumor vessels can be explained by their chaotic architecture that increases their fragility and retards blood flow within the pro-thrombotic tumor milieu [11]. Early VTP approaches required photosensitizer preaccumulation within endothelial cells, leading to damage and impaired endothelial cell function upon illumination. This approach, based on light-activated VDAs, has been clinically applied for the treatment of age-related macular degeneration (AMD) [12] and for the treatment of prostate cancer in animal models [13], [14]. Unfortunately, these protocols demonstrated limited therapeutic potential and rapid extravagation of the circulating photosensitizers into adjacent tissues, with significant consequential lateral damage [12], [13], [15]. Moreover, recent studies showed that peripheral tumor blood vessels (e.g. FAs and DVs) are less sensitive to such VTP approaches [16], consistent with tumor relapse.

We have developed an innovative approach to VTP by applying the novel Palladium-Bacteriochlorophyll derivatives, Tookad [17], [18], [19] and WST11 (Tookad soluble®), as ROS-generating agents [15], [20], [21], [22], [23]. These sensitizers, defined as laser-activated vascular occluding agents (VOA), remain confined within the circulation even at high doses, fail to extravagate to other tissues/organs, and are rapidly cleared by hepatic and renal systems. Thus, Tookad-based photoactivation and ROS generation are intravascular and do not target specific tumor cells or signaling pathways therein. The respective ROS consist of superoxide and hydroxyl radicals, rather than singlet oxygen the prevalent ROS in other VTP approaches [24], [25]. These two species have been reported to initiate pathophysiological processes leading to vessel impairment and occlusion [26], [27], previously unexplored in the context of photodynamic therapy.

Following a single illumination session, treatment success of up to 75% can be achieved, as monitored in luciferase-transfected tumor-bearing mice 24 h post-VTP [21]. Tumor necrosis was observed 24–48 h later, and eradication and healing several weeks thereafter [17], [18], [28]. Tookad-VTP has demonstrated significant clinical efficacy in first- and second-line treatments of patients with localized prostate cancer [29], [30], [31], [32], [33]. WST11-VTP has also proven effective in AMD animal models [15] and its safety has been demonstrated in phase I clinical trials. Furthermore, WST11-VTP has shown promising results in ongoing Phase II clinical trials for the treatment of localized prostate cancer in patients who have been under active surveillance. This minimally invasive single-session protocol, is probably one of the shortest anti-tumor treatment modalities available to date and most importantly, is not associated with significant side effects [32], [34].

The dramatic hemodynamic changes induced within seconds of illumination of Tookad or WST11, lies in sharp contrast to the delayed and temporal vascular arrest (hours/days) observed with other forms of VTP [12], [13], [14], [9]. This difference underscores the hypothesis that Tookad/WST11-VTP-based vessel occlusion involves still unkown and fundamentally different mechanisms.

In this study, a thorough temporal dissection of the vascular response to Tookad-VTP was monitored. The mouse earlobe tumor model was used to enable the follow-up of vascular anatomy and real-time hemodynamic changes during illumination. This model provides easy and non-invasive imaging access to tumor vessels, enabling the three online imaging techniques used: (*i*) fluorescent IntraVital Microscopy (fIVM), (*ii*) online Dynamic Light Scattering Imaging (DLSI) and (*iii*) online photosensitized psMRI [17], [35]. These methods provide complementary, high resolution, temporal information of the blood vessels within and around the tumor.

Here we show that a distinct vasodilatative event, followed by vasoconstriction, blood clot formation and occlusion of the FAs takes place within the first minute of sensitizer illumination. Continued illumination (up to 10 min) resulted in irreversible FA occlusion, while the adjacent normal vessels remained intact or resumed normal function shortly after treatment completion. This rapid and unique response suggests a profoundly novel and effective vascular-occlusion mechanism, outlining the Tookad-VTP-specific targets and mode of action.

## Materials and Methods

### Ethics Statement

All animal experiments were conducted at the Weizmann Institute of Science and approved by the Weizmann Institutional Animal Care and Use Committee (IACUC).

### Animal and tumor models

Cultured MADB106 rat mammary carcinoma cells were s.c. grafted to the earlobe (3•10^5^ per ear) of anesthetized female CD1 nude mice.

### Photosensitizers (PS) and llumination protocol

Tookad® (10 mg/kg, Steba-Biotech, France) was i.v. administered to the tail-vein of tumor-bearing mice. Light (763 nm) was provided by a 1W diode laser (CeramOptec, Bonn, Germany) equipped with an optic fiber with a frontal diffuser (FD1, Medlight S.A, Ecublens, Switzerland), programmed to project a circular uniform light field (diameter  = 11.2 mm) with an average intensity of 150 mW/cm^2^. To illuminate the earlobe target, the light beam was tilted by 20 degrees to enable simultaneous vertical microscopic observation of the tumor forming an elliptical shape. The created gradient of light intensities on the long field dimension, ranged from 155 mW/cm^2^, to 141 mW/cm^2^ on the front and rear edges of the field, (150 mW/cm^2^ +3%/-6%, respectively), while on the shorter dimension the intensity was constant. Uniformity and the gradient intensity slope were verified on a test screen by computerized digital scanning measurements. Considering an average tumor diameter of 3 mm, the variation in light intensity on the tumor surface and the normal surrounding is expected to remain within the acceptable light intensity variance of ±5%. Notably, the threshold light flux for inducing complete tumor vascular occlusion in this animal model and Tookad dose, is 120 mW/cm^2^ (data not shown)

### *In vivo* VTP protocol

Anaesthetized mice were placed on the microscope stage. The animal position and light beam (visualized with an online pointer laser, 3 mW, 630 nm) were aligned for optimal operation. Treatment was initiated with 5 min illumination (Light control, LC), followed by i.v. PS administration under continuous illumination (VTP), and completed 10 min later upon switching the light off. Dark control (PS administration without illumination) was conducted separately. The illuminated region encompassed the entire earlobe and included both the tumor and its healthy surroundings.

### fIVM studies

VTP was conducted on the stage of a zoom upright macroscope (Olympus SZX-RFL2, Japan) equipped with still (AxioCam HRc, Carl Zeiss, GmbH Germany) and video (Mintron 12V1-EX CCD, Taiwan) cameras.

### Online blood flow tracking

Red Blood Cells (RBC) from a donor CD1-nude mouse were stained *ex vivo* with 15 ug/ml 4-(4-(didecylamino)styryl)-N-methylpyridinium-iodide (4-Di-10-ASP, Anaspec, San Jose, CA, USA) for 40 min at room temperature (RT). Free fluorofophore was washed out by centrifugation (500 g, 10 min). Stained RBCs were then i.v. injected (to achieve final ∼1% stained RBC in the recipient mouse blood) shortly before video recording [17]. Leukocytes and platelets were fluorescently stained by i.v. administration of Rhodamine 6G (0.5 mg/kg), as previously described [36], [37], immediately before photosensitizer administration.

### Angiography

I.V.-injected high molecular-weight dextran-FITC (250 kDa) served as a pool marker when using a long pass filter (Ex460-490/Em510 nm). Vasodilatation and vasoconstriction were assessed by subtraction of video images (time  =  t) from that of T_0_ (onset of illumination), defining the net-change in fluorescence at time  =  T_0_+t. Assuming a cylindrical shape, blood vessel volume (BVV) was calculated as BVV = (diameter/2)∧2π * unit length.

### DLSI studies

Dynamic Light Scattering Imaging (DLSI) was performed as described earlier [38] using a custom-made laser illumination ELFI-C unit (10 mW, 670 nm, Elfi-Tech, Rehovot, Israel). As DLSI of tumor blood vessels is based on the temporal contrast of intensity fluctuations produced by light scattered from the illuminated surface, the laser was adjusted to illuminate the mouse earlobe and was imaged through a zoom-upright macroscope (SZX-RFL2, Olympus, Tokyo, Japan) equipped with a PixelFly QE camera (pco. Imaging, Kelheim, Germany).

### MRI studies

The anesthetized mouse was catheterized and its ear was gently attached (double-sided scotch tape) to a Perspex flat carrying plate over the 1.5 cm surface coil. The mouse ear was covered with water-based gel for air isolation. Gradient echo (TR = 100 ms, TE = 10 ms, flip angle of 30°, time resolution of 90 sec) and T_1_w Spin echo (TR = 250 ms, TE = 9 ms) sequences were conducted inside a horizontal 4.7T Bruker-Biospec spectrometer using a transmitter 7.5 cm volume coil and a 1.5 cm receiver-only surface coil. Gd-DTPA (0.1 mmol/kg) was administered via catheter ∼20 min after VTP. All experiments in this study were repeated at least three times and a representative experiment is presented.

### Data analysis

Online video-recordings were analyzed offline using VirtualDub 1.5.10 and ImageJ 1.33u. Light control (LC) blood flow velocities were defined as 100% and time-to-stasis is presented as mean time (min) ± SEM. MRI data processing was conducted with MATLAB 6.5 software, using home-made programs [35]. Positive changes in BOLD contrast were assigned to increases in DeoxyHb levels, while decreased MRI signals were assigned to increases in blood oxygen saturation or in blood flow rates. The percentage of BOLD activation was calculated pixel-by-pixel according to the following equation: [% change  =  (1-image/average of control) ×100], resulting in the activation maps presented below.

### Histopathology

Tissue samples were excised from euthanized mice 24 h after treatment, fixed in 3.7% formaldehyde and underwent standard histological preparation and hematoxylin-eosin staining at the Weizmann Institute Pathology Service Unit.

## Results

### Selective response of the tumor vasculature to Tookad-VTP

**Figure 1:**
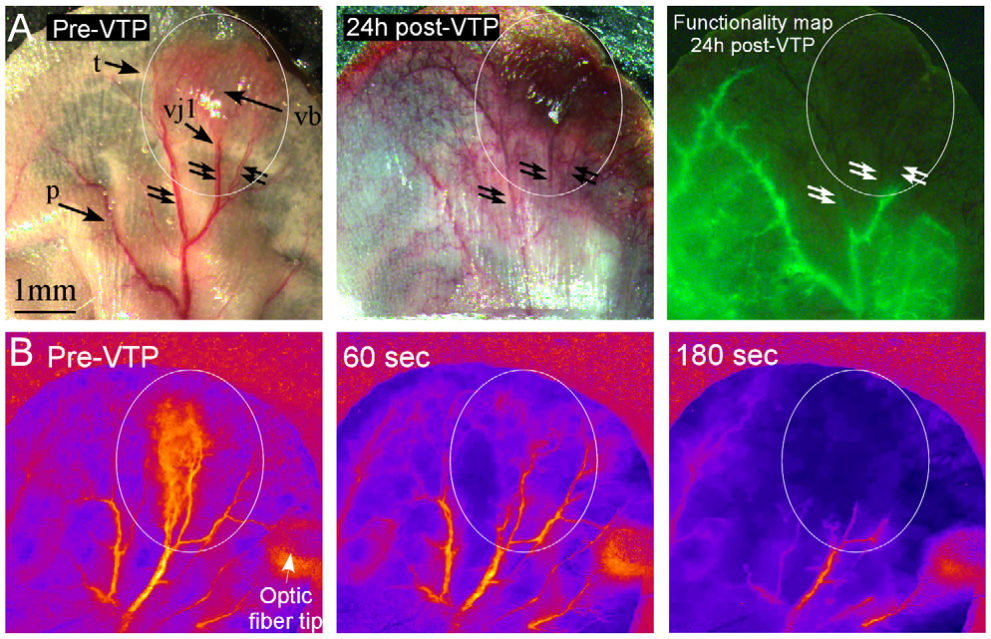
Selective occlusion of tumor vasculature following VTP. A. The tumor (t, marked by an ellipse) containing a discrete vascular bed (vb) is supplied and drained by major FAs and DVs (double arrows). Vascular Junction 1 (vj1) defines the interface between the tumor's small vessels (e.g. capillaries, etc.) and the FAs/DVs. Pre-existing, non-tumor vessels (p) can be seen in the surrounding normal tissue. The same tumor area is depicted in the middle panel 24 h after VTP. The right hand panel is an image captured during angiography follow up at 24 h post-VTP. Selective loss of tumor blood vessel functionality, as seen by exclusion of the fluorescent marker, was apparent, while normal vasculature in the surrounding tissue remained functional. B. Dynamic light scattering imaging of a mouse earlobe before, 60 sec and 180 sec after Tookad-VTP initiation demonstrated selective reduction in tumor perfusion. Towards the end of the treatment (180 sec), no perfusion was detected in the tumor zone, while the surrounding vasculature remained functional until treatment completion. Images were produced by temporal contrast calculations and are presented as color-scale coded maps (higher perfusion is illustrated as lighter colors). These images are clips selected from Video S1. A and B are represented by two individual animals. All other details are described in the Materials and Methods section.

Following implantation, distinct adaptation of the earlobe's preexisting vascular network transpired, in support of angiogenesis-dependent tumor development. Newly formed FA and DV, functioning as both the portal for inflow and drainage of the tumor microcirculation were clearly identifiable (double arrows in Figure 1A). This newly formed network underwent a gross response to Tookad-VTP (Figure 1A), where signs of edema and changes in blood vessel morphology were evident in and immediately around the tumor (Figure 1A, middle panel) within twenty-four hours of Tookad-VTP. In addition, significant losses in blood vessel functionality were recorded by Dextran-FITC angiography. Blood flow arrest was visible in the FAs and DVs, as evident by absence of the fluorescent marker (Figure 1 Right panel). Arterioles and venules of similar size and hierarchy in the adjacent, healthy tissue appeared functional, despite their concomitant subjection to the complete VTP protocol.

Specificity of the tumor vascular response to VTP was further demonstrated by the real-time changes in vessel functionality, as monitored by DLSI (Figure 1B). Here, the selective decline in tumor perfusion was observed with the progress of Tookad-VTP. Towards the end of the first minute of illumination, perfusion ceased in the tumor vessels, while most of the surrounding, healthy vasculature remained functional until the end of the recording (5 min, Figure 1B and Video S1).

**Figure 2:**
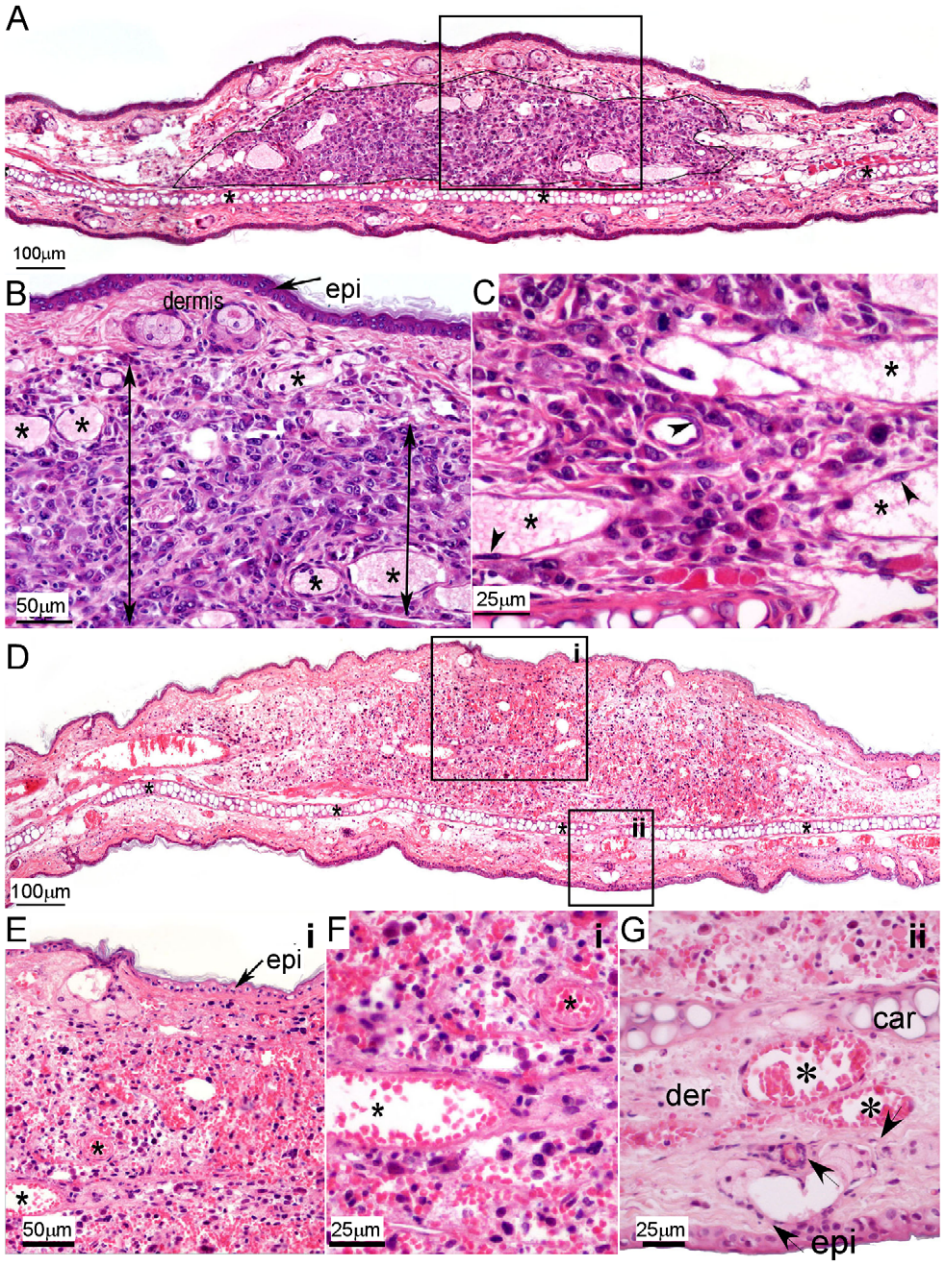
Histopathology of the Tookad-VTP-treated tumor-bearing earlobe. Hematoxilin-Eosin staining of untreated MADB106 tumor-bearing ear sections (A–C) compared to Tookad-VTP-treated tumors 24 h post treatment (D–G) are shown. A. (magnification: ×10) The untreated tissue included viable neoplastic cells which formed a small and well-demarcated mass (outlined in black) within the dermis of the pinna. The aural cartilage is identified by asterisks. The boxed area is further magnified in B (x20) and includes the epidermis (epi), dermis, and the lumen of several vascular spaces (asterisks). At higher magnifications (C, ×40) several blood vessels surrounded by neoplastic cells were identified (lumen identified with asterisks), where the endothelial cells lining them (arrowheads) were shown to be viable. D. (magnification: ×10) 24 h following Tookad-VTP, necrosis, hemorrhage, edema and vascular congestion in the tumor area were apparent (boxed in i), with only a small degree of similar findings in the surrounding tissues (boxed in ii). E. Magnified (x20) tumor area of (Di) showed diffuse necrosis and hemorrhage, no identifiable viable neoplastic cells and a necrotic epidermal layer (epi) above the tumor. The lumen of blood vessels is marked (asterisks). F. Further magnification (x40) showed the diffuse necrosis which affected all cells and included extravasated red blood cells indicating acute hemorrhage. Two blood vessels were identified (asterisks) but no viable endothelial cells were seen. G. Magnified (x40) surrounding tissues of (Dii) showed necrotic neoplastic tissue located above the aural cartilage (car). The dermis below the cartilage (der) was mildly edematous and contained dilated dermal blood vessels (asterisks). Epidermal cells (epi) and sebaceous gland cells (arrows) contained viable nuclei.

Histopathology of the treated earlobe/tumor at 24 h post-treatment, demonstrated tumor necrosis accompanied by hemorrhage, edema and vascular congestion in the tumor and tissues within a 1–2 mm radius of the treated tumor margin. In contrast, normal tissues at larger distances, but still exposed to the full treatment protocol, remained viable and intact (Figure 2). These results suggest heightened susceptibility of the tumor vasculature including FAs and DVs, to Tookad-VTP.

### VTP-induced hemodynamic alterations in the tumor blood flow

**Figure 3:**
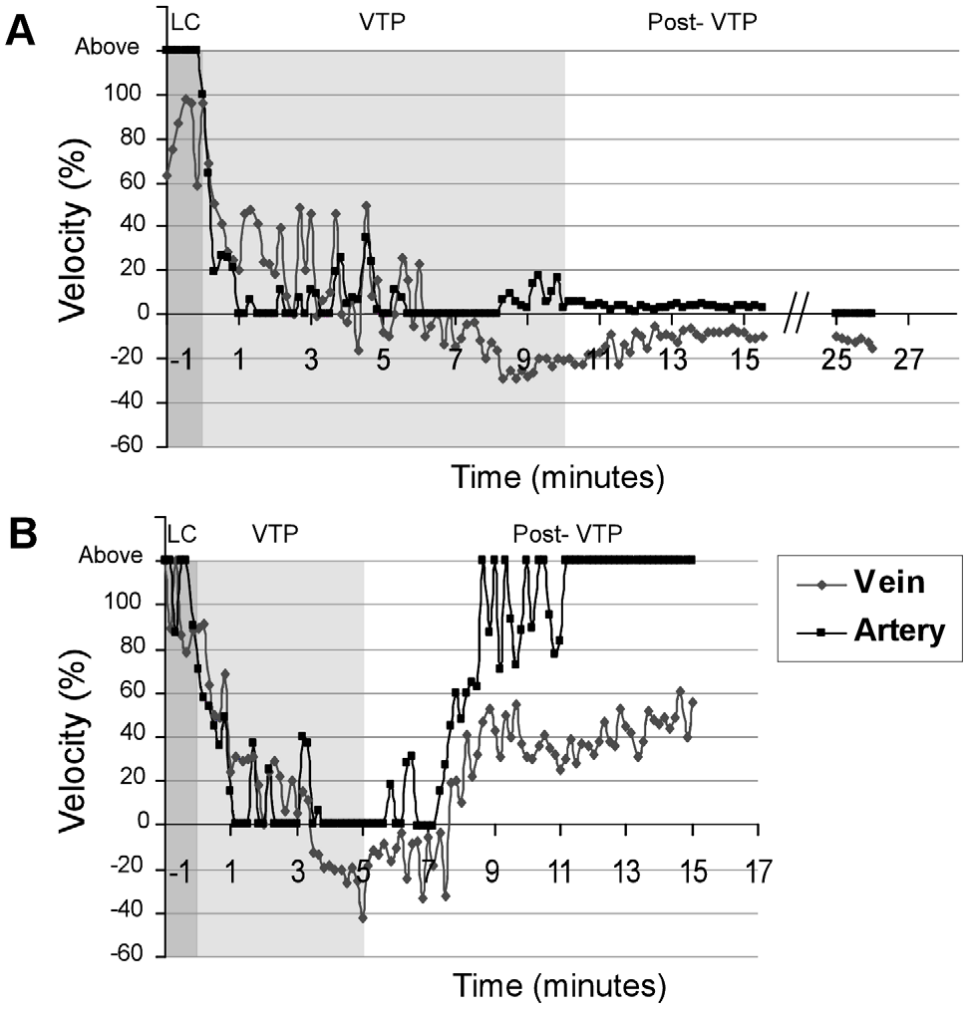
VTP-induced changes in tumor blood-flow. Arterial and venous blood flow during the last minute of light control (LC, dark-gray background), through the full 10 min VTP protocol (A, light-gray background, n = 3, representative mouse is shown) or partial, 5 min treatment protocol (B, light-gray background, n = 3, representative mouse is shown) were monitored. Time resolution = 10 sec. Baseline (100%) was defined by the average blood flow value during the 5 min LC subsession. A sharp decline in the FA flow rate, followed by its collapse after ∼1 min VTP, was accompanied with random pauses and reversed venous blood flow (represented as negative flow values). The standard VTP protocol induced irreversible arterial blood flow arrest, with a concomitant reversal in venous blood flow direction (A), observed to persist until the end of data acquisition. Early termination (light off at 5 min) led to the rehabilitation of blood flow levels (B).

fIVM was applied during Tookad-VTP in attempt to characterize changes in blood flow within FAs, DVs and the tumor microvasculature. To this end, RBCs from a donor mouse were fluorescently stained *ex vivo* and i.v. injected to the recipient mouse shortly before the video recording. RBC monitoring demonstrated blood flow arrest in the superficial tumor microvasculature within the first minute of illumination and did not resume throughout the remainder of the monitoring session (Video S2). Blood flow changes in the monitored FA and DV within the illuminated region, defined as ‘Vascular Junction 1’ (Video S3), showed a remarkably different pattern; Flow velocities quantified offline at a 10 sec time resolution (Figure 3), demonstrated a sharp decline in FA flow rate at the onset of VTP, and reached perfusion arrest at a mean 63.2±1.5 seconds (n = 5) (Figure 3A). Following a short delay (seconds), intermittent FA blood-flow was observed for 5–6 min, characterized by irregular spikes and pauses that were almost simultaneously reflected its paired DV. At t_VTP_>5 min, irreversible FA blood flow arrest, coupled with a reversal in DV blood flow direction was observed in 100% of the monitored cases (Figure 3A) and remained as such until the end of the data acquisition period (15–30 min post-VTP). Some normal vessels within the illuminated domain occasionally underwent constriction as well, but regained functionality shortly after treatment ending (range of minutes). Early treatment termination (t_VTP_ = 5 min) led to complete and partial rehabilitation of the FA and DV blood-flow, respectively, without sustained reversal in venous blood-flow direction (Figure 3B).

Thus, the hemodynamic FA response pattern to VTP is comprised of at least two distinct phases. The early response phase includes FA occlusion within ∼60 sec of VTP, but is reversible if the treatment is not completed. The second phase involves complete and irreversible tumor vasculature collapse that lasts beyond treatment completion.

**Figure 4 pone-0010282-g004:**
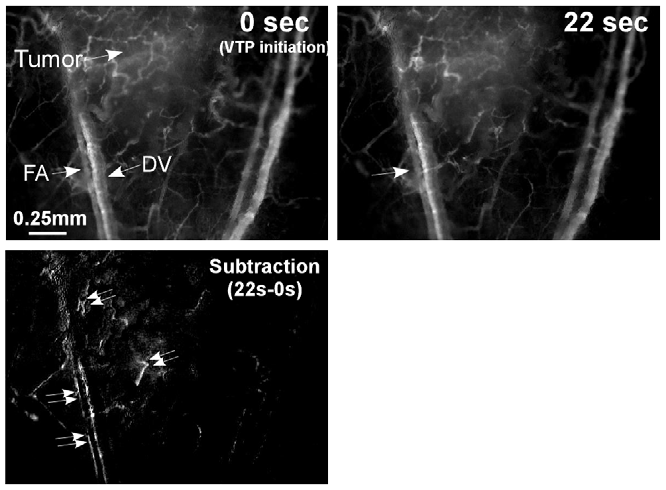
Tookad-VTP induced changes in blood vessel diameter and morphology. Angiography images of the illuminated area, extracted from Video S4 before treatment and at t_VTP_ = 22 sec are presented. Subtraction of the latter from the former is shown in the lower image. Arrows indicate FAs/DVs. An increase in the vascular-associated fluorescence (indicated by double-arrows), peaking at 22 seconds from the onset of illumination was observed within the tumor boundaries, but not in the arteries that perfused the surrounding normal tissue.

**Figure 5 pone-0010282-g005:**
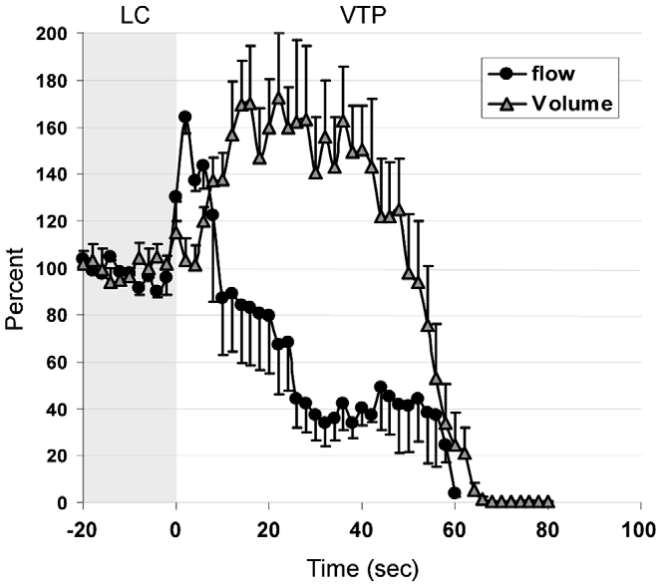
Tookad-VTP induced increase in arterial blood flow rate and volume. Arterial blood flow rates and volumes were monitored in the tumor FA during VTP. Baseline (100%) was defined by the average of LC recordings (gray background). Data analyzed at a 2 sec time resolution is presented as the mean ± SEM (n = 5). A prompt, but transient ≤60% increase in arterial blood flow velocity, followed by an up to 70% elevation in FA blood volume was observed upon initiation of VTP. Approximately twenty seconds later, a gradual decline in the arterial blood volume and flow rate was observed to the point of complete vascular collapse, whereby stasis was observed at t_VTP_≅60 seconds.

To further explore the prompt FA response and the early-phase hemodynamic parameters, angiography of the treated area was conducted throughout illumination. An increase in the vascular-associated fluorescence, peaking at 22 seconds from onset of illumination was observed within the tumor boundaries, but not in the arteries that perfused the surrounding normal tissue (Figure 4 and Video S4). Alterations in FA vessel diameter, as extrapolated from angiography signals (expressed as percent changes in blood volume) and blood flow rates within the FA were then plotted as a function of time (Figure 5). A transient increase in FA blood flow velocity, peaking at 160% of pre-illumination rates, was observed 2–6 sec after VTP initiation (n = 5) and followed by an up to 170% elevation in arterial blood volume that peaked ∼20 seconds after VTP initiation. This phase was then followed by gradual declines in arterial blood volume and flow rates until vascular shutdown at 60 seconds. Cumulatively, these results suggest that FAs comprise the primary targets of Tookad-VTP. No significant vasodilatation was observed in the normal vasculature while, as mention above, vasoconstriction, if observed, was typically transient.

### Rapid blood clot formation during Tookad-VTP

**Figure 6:**
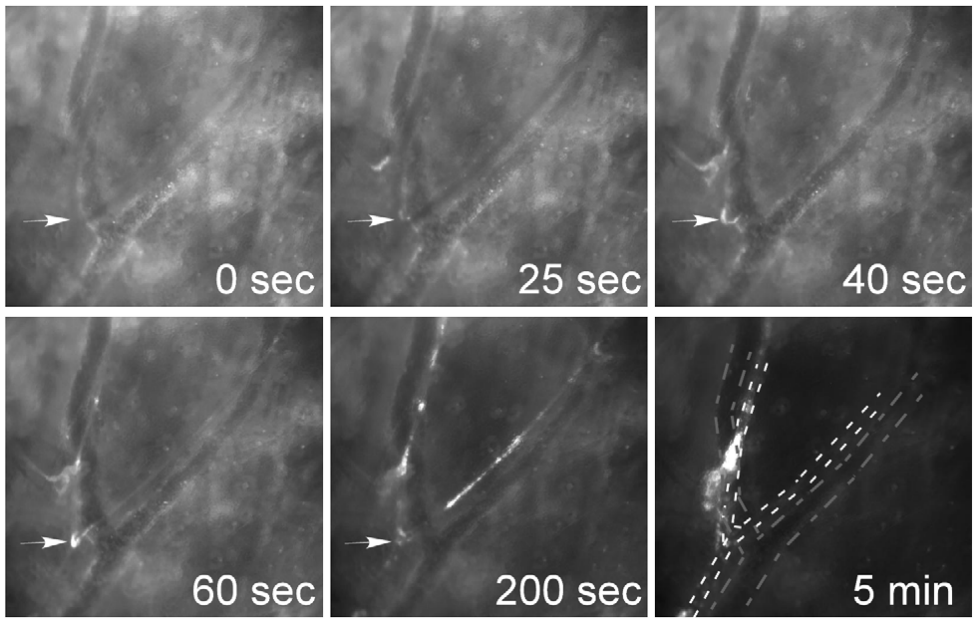
Tookad-VTP induced blood clotting. Circulating leukocytes and platelets were stained *in vivo* with Rhodamine6G immediately before sensitizer injection and were monitored online throughout the full VTP protocol (images taken from Video S5). Blood clot formation began on the inner vessel wall within 25 sec of VTP initiation, particularly at the vessel bifurcation points, and was concomitant with vessel constriction. Five minutes after VTP onset, a newly formed blood clot occluded the FA, and significantly obstructed tumor arterial blood supply. White and gray dashed lines define arterial and venous boundaries respectively.

VTP-induced hemodynamic changes, viewed within the FAs during the early phases of treatment, were accompanied by blood clot formation and a gradual increase in the microvasculature permeability. In order to directly examine vascular clot formation, circulating leukocytes and platelets were stained *in vivo* with Rhodamine6G immediately before sensitizer injection, allowing for online imaging of fluorescently-labeled blood clots (Video S5). Formation of blood clots on the inner FA walls, particularly at pre-capillary bifurcation points, was observed within 25 sec of VTP onset (Figure 6), followed by blood clot mobilization towards the interface of the tumor microcirculation, leading to subsequent vessel occlusion.

### BOLD contrast changes during VTP as viewed by psMRI

**Figure 7:**
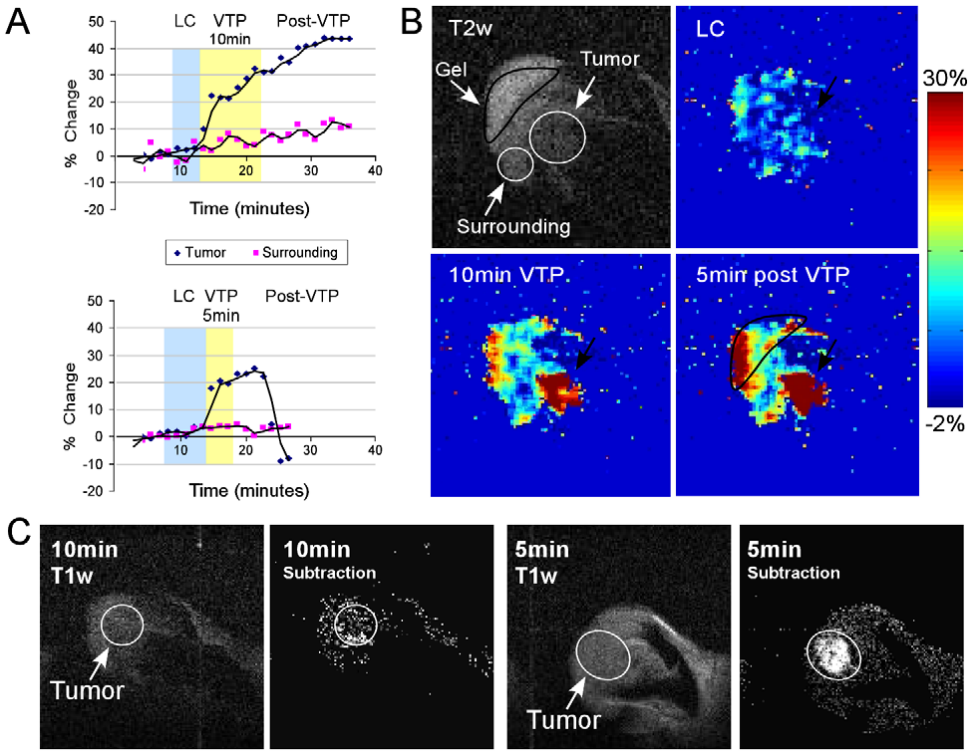
VTP-induced changes as seen via BOLD contrast. A. Graphic presentations of percent changes in BOLD contrast before treatment, during LC (blue background), 10 min-VTP or 5 min–VTP (yellow background – upper and lower graphs, respectively) and after VTP are shown. Increases in BOLD contrast signals upon VTP remained constant when illumination continued for 10 min, but declined to pretreated values when illumination was terminated at 5 min. B. T2w image of the ear tumor, normal surrounding tissues and gel is presented. BOLD activation maps of LC, 10 min VTP and 5 min post-VTP are presented (images taken from Video S6). C. Spin-echo T1w images following 10 min or 5 min-VTP are shown. Gd-DTPA was administered ∼20 min post-VTP. The T1w post-injection images were subtracted from the pre-injection images and the difference are shown (2^nd^ and 4^th^ panels). Exclusion of Gd-DTPA following standard 10 min VTP confirmed tumor-blood stasis, while perfusion of Gd-DTPA following the partial 5 min illumination indicated blood-flow rehabilitation.

Fluorescent microscopy-based, online assessment of VTP-induced hemodynamic changes offers visualization depth that is limited to the peripheral tissue surface. To overcome this limitation, we applied psMRI, enabling imaging at practically any tissue depth [17], [35]. Changes in BOLD contrast in the treated area, as detected online by psMRI, paralleled those monitored using fIVM techniques (Figure 3). No changes in BOLD contrast values were observed before treatment onset or during illumination alone (LC) (Figure 7A). However, the onset of VTP induced a prompt increase in BOLD contrast signals exclusively in the tumor area, which continued to rise throughout the illumination phase and peaked at 30% above baseline values (Figure 7A, n = 6). Upon completion of the treatment protocol, BOLD contrast signals further intensified, leveling off at >40% above initial signals towards the end of data acquisition (Figure 7A, upper graph, Figure 7B and Video S6). These results comply with a vascular accumulation of deoxyHb, signifying photo-consumption of oxygen, a reduction in blood-flow rates due to reduced inflow of fresh oxygenated blood, and reversed flow of deoxyHb-enriched venal blood. Vascular functionality in the target area was measured 20 min post-VTP, by dynamic contrast-enhanced MRI. A loss of vessel functionality was apparent following the complete VTP protocol (Figure 7C, 10 min). In contrast, upon early termination of VTP (t_VTP_ = 5 min), a resumption of oxygenated blood-flow was indicated by a return of BOLD contrast values to pre treatment values and below (Figure 7A, lower graph and Video S7). In addition, the vasculature was found to be fully functional 20 min post-VTP (Figure 7C, 5 min). No similar hemodynamic changes were observed in the surrounding tissues. Thus, VTP-associated BOLD contrast signals tightly correlated with and complemented the hemodynamic changes measured by both fIVM and DLSI.

## Discussion

The advantage of VTP over other systemically administered anti-vascular approaches lies in its highly localized toxic effects, as defined by the illuminated region. Tookad-VTP differs from other VTP modalities in five critical aspects: (1) the sensitizer does not extravasate from the circulation and, therefore, the impact of VTP is confined to the vascular lumen regardless of the drug/light interval; (2) superoxide and hydroxyl radicals, rather than singlet oxygen, are the key mediators of its photodynamic effect, consequently inducing a different chemistry of damage; (3) the prompt ROS impact is within the circulation, and not in vascular endothelial cells; (4) induction of irreversible vascular collapse and blood stasis in the tumor is accomplished within a few minutes of illumination, [17], [18] and primarily involves the FAs and DVs at the tumor rim, and (5) in contrast to other vascular disruptive approaches, Tookad-VTP achieves tumor ablation as a mono-therapy, involving initiation of ischemic necrosis with secondary radical formation as reported in preclinical and clinical studies [18], [32].

In this study, the details of the swift hemodynamic tumor vasculature response to Tookad-VTP were examined by three complementary, real-time imaging methods. These techniques allowed for identification of the affected blood vessels and sequential delineation of the events leading to tumor blood stasis. Tumor-FAs comprised the primary target of Tookad-VTP (Figures 1 and 6), where blood supply to the tumor microcirculation was promptly arrested by local blood clots accumulating at the tumor FA-capillary interface (Figures 6 and Video S5).

In addition, the response of the tumor vessels to ROS assault involved prompt and significant FA vasodilatation (Figures 4–5) with subsequent constriction, blood clotting and vessel obstruction (Figures 3–6), culminating in irreversible flow arrest at illumination times ≥5 min (Figure 3). The extensive FA and DV vasoconstriction described for Tookad-VTP, were not observed in other VTP modalities [39] and are suggested to be critical in their irreversible occlusion. Absence of contractile smooth muscle elements in tumor vasculature deem it unlikely that tumor capillaries actively participate in vasodilatation or vasoconstriction processes. Such activity is more consistent with that of the larger, more developed FAs and DVs (Figures 4–6). The susceptibility of these vessels to Tookad-VTP was found to be highly selective, when compared to their adjacent, healthy counterparts that remained functional, despite exposure to the same destructive procedure.

The driving force behind transient FA vasodilatation at the onset of Tookad-VTP is believed to be tightly related to the abrupt jump in oxygen consumption, coupled with ROS generation [35], [40]. In efforts to restore local normoxia, the hypoxic vasodilatative state triggers reperfusion of the illuminated area with oxygenated blood [41], [42]. Instantaneous, local release of nitric oxide (NO•), a potent vasodilator, from intravascular pools [43], [44], [45] is believed to play a key role in this process, facilitating the VTP-induced burst in arterial blood flow (Figures 4, 5 and Video S4). Moreover, the generated NO• may further interact with the photogenerated superoxide and hydroxyl radicals and consequently play a critical role in the pathophysiological developments (Gal, Y and Madar-Balakirski, N et. al. 2010, in preparation), [46], [47], [48] towards rapid vascular occlusion. Interestingly, when BOLD MRI was recorded in a different tumor model, a brief, transient signal dip preceded the elevation of BOLD contrast. This phenomenon was observed within the time frame of the induced hypoxic vasodilatation (Figure 4), and is likely related to the transient and short blood-flow burst and re-oxygenation process (Tempel-Brami et al. 2010, in preparation).

The massive and almost instantaneous blood clot formation observed in FAs (Figures 6, S4 and S5) appears markedly different from the thrombus formation observed following VTP with other agents, proposed to be the consequence of the physical vascular damage. Extreme vasodilatation and constriction can cause dramatic changes in local flow conditions, significantly affecting the transport of molecules to and from the vessel wall. They can also lead to alternating flow directions within tumor vessels, as was already described two decades ago [10], [49]. Such activity can induce platelet, erythrocyte and white cell activation, protein redistribution and accelerated cell aggregation [50]. Blood clots formed shortly after illumination primarily blocked down stream flow in FA, subsequently preventing the blood flow from reaching the unobstructed capillaries (Figure 6). In the absence of arterial blood supply, a reversal in DV flow back into the tumor microcirculation is expected, and was indeed observed (Figure 3).

By providing a means to directly monitor vascular oxygen levels, with no limits to tissue depth, psMRI monitoring of Tookad-VTP-induced responses complemented the findings obtained by the fast-capture imaging techniques applied. The increase in BOLD contrast observed within the tumor boundaries upon initiation of VTP (Figure 7A–B), correlated with photochemical oxygen consumption, decreased arterial flow rates (Figures 3, 5) and blood clot formation (Figure 6). However, despite complete arterial stasis, sustained increases in post-VTP BOLD contrast signals were noted in the bulk of the tumor tissue. The timeline of this increase correlated well with that of the reversed deoxyHb-rich venous blood flow into the tumor (Figure 3A). Reversed blood flow from the draining veins into the tumor was described earlier by others and said to reflect the impaired development of the tumor vasculature [10]. However, such flow back into the tumor capillaries suggests they are still open and remain functional long after full FAs occlusion (Figures 3, 6). The time course of the BOLD signal also indicates complete oxygen depletion in the tumor capillaries after >5 minutes of illumination, a significantly longer interval than that required for the occlusion of the FA (Figure 7).

Cumulatively, this study marks tumor FAs as the primary target of Tookad-VTP. Their rapid occlusion leads to subsequent tumor ablation via a necrotic process, as described by us previously. The initial success of Tookad-VTP as a mono-therapy for localized prostate cancer [31], [34] is intriguing and strengthens our mechanistic claims.

## Supporting Information

Video S1 Dynamic light scattering imaging of the mouse earlobe during Tookad-VTP. In this video clip, the images of the mouse ear blood vessel were produced after temporal contrast calculations, as previously described (Kalchenko V, Preise D, Bayewitch M, Fine I, Burd K, Harmelin A. *In vivo* dynamic light scattering microscopy of tumour blood vessels. J Microsc. 2007;228:118–122) and presented as a color coded map (higher perfusion is illustrated by lighter colors). Normal perfusion is apparent throughout the untreated control (−120 sec to −60 sec) and light control (−60 sec to 0 sec) periods. Tookad was injected at time zero. With progression of Tookad-VTP, a selective and gradual reduction in tumor perfusion was observed (60 sec). Towards the end of the treatment session (200 sec), absence of perfusion was detected in the tumor zone alone, while the surrounding vasculature remained functional throughout. All other details are described in the Materials and Methods section.
(5.90 MB AVI)

Video S2 Blood flow in the tumor vascular bed during Tookad-VTP. Blood flow in the tumor vascular bed was monitored in real-time using injected fluorescently-labeled erythrocytes (4-Di-10-ASP) from donor mice. Blood flow in tumor surface vessels stopped shortly after onset of VTP and did not resume post-VTP (6.5 minutes) and up to the end of recording session (30 min, data not shown).
(3.15 MB AVI)

Video S3 Blood flow in the tumor's FA and DV during Tookad-VTP. Blood flow was monitored in real-time using injected fluorescently-labeled erythrocytes (4-Di-10-ASP) from donor mice. No significant changes in flow were detected during the light control phase. Soon after VTP onset (time counter icon turns red), blood flow velocity in the main vessels decreased, until full vascular collapse at ∼60 sec. At t_VTP_ = 2 min, FA flow still had not recovered and DV blood velocity dramatically decreased, while the vessels in the surrounding tissue (also exposed to light and drug) showed normal behavior. Reversed venous blood flow was evident at t_VTP_ = 5 min. Selective, irreversible FA blood stasis and reversed venous blood flow remained until the end of data acquisition period, 30 min post-VTP onset.
(2.66 MB AVI)

Video S4 Tookad-VTP induced changes in vessel morphology. Angiography of the illuminated area (250-kDa dextran-FITC) during Tookad-VTP reveals morphological changes primarily in the tumor boundaries. With progress in VTP, gradual increase in vascular permeability of the tumor vessels (manifested by extravasation of the fluorescent pool marker) indicates vessel wall damage. The observed dark shadows in the tumor microvasculature are attributed to blood clots that exclude and shield the pool marker fluorescence. Towards protocol completion (the end of the movie) tumor vessels appear collapsed.
(4.45 MB AVI)

Video S5 Tookad-VTP induced blood clotting. Circulating platelets and leukocytes were stained *in vivo* with Rhodamine 6 G immediately before sensitizer injection. FA and DV at “vascular junction 1” were monitored before and during Tookad-VTP. Formation of blood clots on the inner FA walls, particularly at pre-capillary bifurcation points was observed soon after VTP onset (t_VTP_ = 17 sec), and correlated with vessel constriction (t_VTP_∼34 sec, peaking at t_VTP_∼60 sec). This was followed by mobilization of blood clots towards the interface of the tumor microcirculation with subsequent vessel occlusion.
(1.44 MB AVI)

Video S6 Changes in BOLD contrast during Tookad-VTP. Change in BOLD contrast during VTP is presented as a sequence of color-coded activation maps. (Intensity bar is defined in Figure 6). Upon VTP onset (digits turn red), a sharp increase in BOLD contrast was observed in the illuminated tumor zone, while signal changes in the normal, surrounding tissues were much smaller (increase in contrast levels at the ear margins was due to gel dryness). Termination of the light at t_VTP_ = 10 min did not lead to a reduction in BOLD contrast signals. Time resolution of each frame is 90 sec.
(0.61 MB AVI)

Video S7 Changes in BOLD contrast during the suboptimal Tookad-VTP protocol. Change in BOLD contrast signals during VTP is presented as a sequence of color-coded activation maps. (Intensity bar is defined in Figure 6). Upon VTP onset (digits turn red), a sharp increase in BOLD contrast signals was observed in the illuminated tumor zone. Termination of the light at t_VTP_ = 5 min (suboptimal treatment) led to a decline in contrast levels to their pretreated values and even below. Changes in BOLD contrast in the normal surrounding tissues were much smaller (increase in contrast levels at the ear margins was due to gel dryness). Time resolution of each frame is 90 sec.
(0.73 MB AVI)
